# Effects of focused ultrasound in a “clean” mouse model of ultrasonic neuromodulation

**DOI:** 10.1016/j.isci.2023.108372

**Published:** 2023-10-31

**Authors:** Hongsun Guo, Hossein Salahshoor, Di Wu, Sangjin Yoo, Tomokazu Sato, Doris Y. Tsao, Mikhail G. Shapiro

**Affiliations:** 1 Division of Chemistry and Chemical Engineering, California Institute of Technology, Pasadena, CA 91125, USA; 2 Division of Engineering and Applied Science, California Institute of Technology, Pasadena, CA 91125, USA; 3 Division of Biology and Biological Engineering, California Institute of Technology, Pasadena, CA 91125, USA; 4 Department of Molecular and Cell Biology, University of California, Berkeley, Berkeley, CA 94720, USA; 5 Howard Hughes Medical Institute, Pasadena, CA 91125, USA

**Keywords:** Optical imaging, Biological sciences, Neuroscience

## Abstract

Recent studies on ultrasonic neuromodulation (UNM) in rodents have shown that focused ultrasound (FUS) can activate peripheral auditory pathways, leading to off-target and brain-wide excitation, which obscures the direct activation of the target area by FUS. To address this issue, we developed a new mouse model, the double transgenic Pou4f3^+/DTR^ × Thy1-GCaMP6s, which allows for inducible deafening using diphtheria toxin and minimizes off-target effects of UNM while allowing effects on neural activity to be visualized with fluorescent calcium imaging. Using this model, we found that the auditory confounds caused by FUS can be significantly reduced or eliminated within a certain pressure range. At higher pressures, FUS can result in focal fluorescence dips at the target, elicit non-auditory sensory confounds, and damage tissue, leading to spreading depolarization. Under the acoustic conditions we tested, we did not observe direct calcium responses in the mouse cortex. Our findings provide a cleaner animal model for UNM and sonogenetics research, establish a parameter range within which off-target effects are confidently avoided, and reveal the non-auditory side effects of higher-pressure stimulation.

## Highlights

• A “clean” mouse model minimizes off-target auditory effects of UNM
• Increasing FUS pressure induces thermal and/or non-auditory confounds
• Intense FUS elicits spreading and destructive brain depolarization
• Direct cortical activation is not observed with the tested FUS parameters

## Graphical abstract

**Figure:**
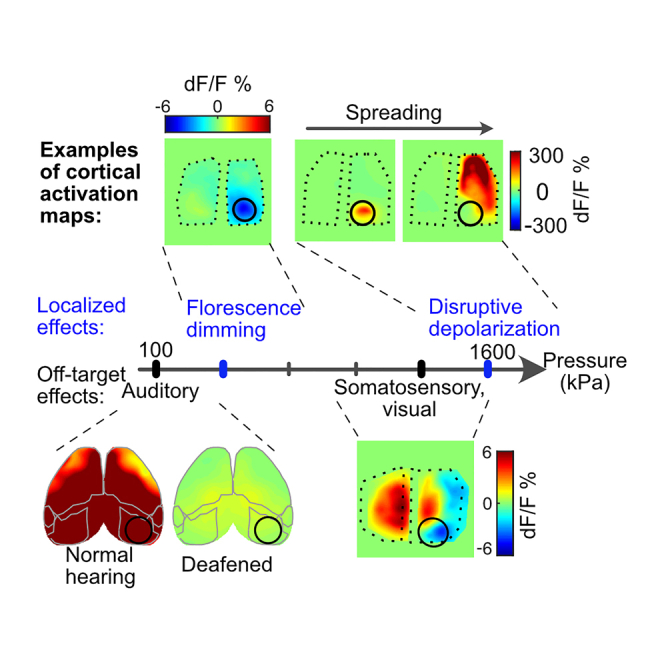

## Introduction

Focused ultrasound (FUS) has the potential to modulate cortical and deep brain regions with a spatial resolution on the scale of millimeters, considerably more precise than established non-invasive neuromodulation technologies such as transcranial magnetic stimulation and transcranial current stimulation.^1^^,^^2^^,^^3^^,^^4^^,^^5^^,^^6^^,^^7^^,^^8^^,^^9^^,^^10^^,^^11^^,^^12^^,^^13^^,^^14^^,^^15^^,^^16^^,^^17^^,^^18^^,^^19^ Multiple physical effects of FUS, such as mechanical force, heating, and cavitation, have been proposed as the underlying cellular and molecular mechanisms of ultrasonic neuromodulation (UNM).^20^^,^^21^^,^^22^^,^^23^^,^^24^^,^^25^^,^^26^^,^^27^ A recent detailed biophysical study in cultured cortical neurons found that the mechanical effects of FUS excite neurons via specific mechanosensitive ion channels.^28^ However, in live rodents, FUS can activate peripheral auditory pathways and cause off-target activation throughout the brain, including both ipsilateral and contralateral regions, regardless of the specific brain targets being stimulated, presenting a persistent challenge in UNM experiments in rodents.^29^^,^^30^ In UNM human studies, subjects have also reported hearing audible tones, where the detection rates can be reduced by auditory masking.^31^^,^^32^^,^^33^ To eliminate these confounds in rodents, using deaf animal models is an effective approach. However, surgical or chemical methods of deafening can be invasive or cause systemic toxicity, which limits the potential for fully awake and long-term chronic experiments.^29^^,^^30^^,^^34^ A recent UNM study used genetically deaf knockout mice with deficits in the inner hair cells,^35^ but their congenital deafness may impair brain plasticity and cortical development, potentially limiting their utility in experiments involving sensory processing, learning, and other cognitive functions.^36^^,^^37^ Therefore, it remains challenging to non-surgically deafen adult normal hearing mice without compromising their capability for neural recording and behavioral experiments.

Here we address this challenge by developing a new “clean” mouse model for ultrasound (US) research and characterizing the safe parameter range for future UNM and sonogenetics studies. In contrast to a knockout mouse model, the double transgenic mouse model (Pou4f3^+/DTR^ × Thy1-GCaMP6s) does not inactivate specific genes expressed in the brain. Instead, it has human diphtheria toxin receptor (DTR) placed downstream of the Pou4f3 promoter, sensitizing hair cells to diphtheria toxin (DT), which allows us to use DT to rapidly, non-invasively, and selectively ablate all the hair cells without causing systemic toxicity to the mice at any point in life.^38^ These mice will maintain normal hearing after birth, which promotes normal brain development and potentially makes them appropriate candidates for conducting high-level behavior experiments. Additionally, other genetically deaf knockout mouse models did not express fluorescence in the brain.^35^ In contrast, our mice express calcium indicator GCaMP6s in the brain, enabling us to simultaneously read neuronal activity across different cortical regions in awake, deafened mice during UNM.

Using this new mouse model, we show that FUS-elicited auditory confounds and off-target brain activation can be effectively reduced or eliminated up to certain pressures in fully awake, deafened mice. As the pressure was increased, the fluorescence signal at the FUS focus gradually decreased due to focal heating and the fluorophore’s thermal dependence. Additionally, high-pressure FUS elicited bilateral off-target brain activation from non-auditory brain regions, possibly due to the activation of non-auditory peripheral sensory receptors. When the pressure exceeded 1,600 kPa, we observed strong calcium responses originating from the FUS focus and subsequently propagating throughout the ipsilateral hemisphere. This strong depolarization was associated with brain damage, as confirmed by histology, indicating that high-pressure FUS causes damage-evoked seizure-like spreading depolarization. These findings provide key insights for future UNM and sonogenetics studies. First, they offer an effective deaf mouse model where the sensory confounds of UNM can be eliminated up to a certain pressure. Second, they delineate an FUS parameter space where non-auditory confounds and tissue damage can be minimized. Third, with thermal fluorescence dimming, they provide a convenient approach to visualize the focus of FUS in the cortex.

## Results

### Diphtheria toxin deafens double transgenic Pou4f3^+/DTR^ x Thy1-GCaMP6s mice

**Figure 1:**
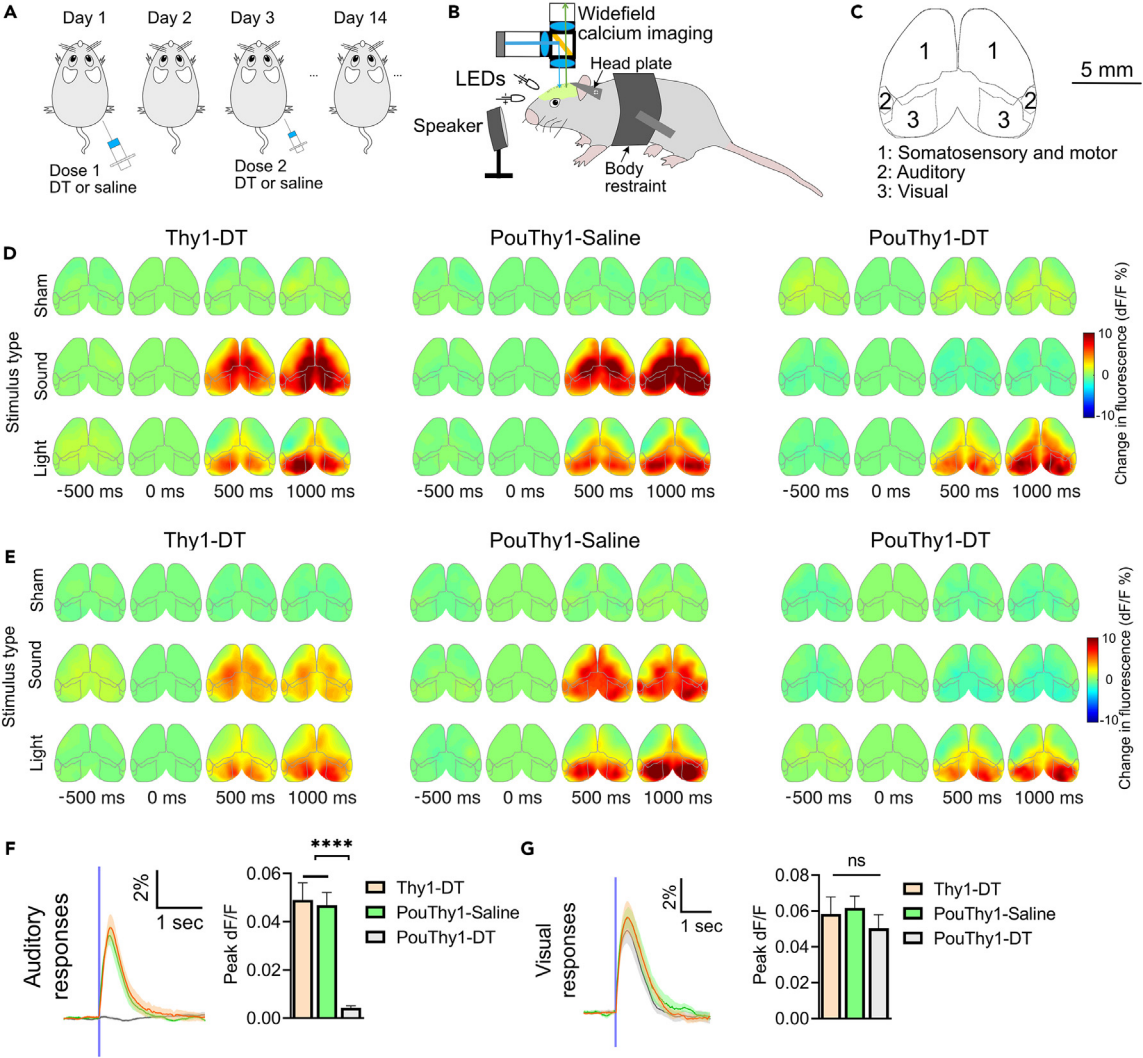
Cortical responses to sham, audible sounds, and light flashes (A) Diagram of protocol for mouse deafening. Two injections of diphtheria toxin (DT) or saline were spaced two days apart. Wide-field imaging experiments were performed at least two weeks after the first injection to wait for the ablation of hair cells in the cochlear and utricles. (B) Illustration of wide-field calcium imaging setup. A speaker was positioned in the front of the mouse. Two LEDs were positioned to the right and left eyes, respectively. (C) Illustration of the top view of the cortex from Allen Mouse Brain Common Coordinate Framework (CCFv3). The visual, auditory, somatosensory, and motor cortices were indexed with numbers. (D and E) Representative examples of cortical activation map to sham, light flashes to both eyes, audible broadband noises to both ears. Two animals are presented for each group. In the sham trials, no stimulus was presented or applied. (F) Auditory responses to audible sounds and the peak dF/F of the Thy1-DT, PouThy1-saline, and PouThy1-DT groups (n = 10 mice for each group, one-way ANOVA ∗∗∗∗p < 0.0001, Tukey’s post comparison). The onset time of stimulation is shown as a vertical blue line. (G) Visual responses to light flashes and peak dF/F of visual responses of the Thy1-DT, PouThy1-saline, and PouThy1-DT groups (n = 10 mice for each group, one-way ANOVA p = 0.1050, ns is not significant). Mean trace is solid and SEM is shaded. Bar graph values represent mean ± SEM.

Transgenic Pou4f3^+/DTR^ (Pou) mice were bred with Thy1-GCaMP6s (Thy1) mice to produce double transgenic Pou4f3^+/DTR^ × Thy1-GCaMP6s (PouThy1) mice, expressing the heterozygous human DTR from the endogenous Pou4f3 locus,^39^ and the fluorescent calcium indicator GCaMP6s.^40^ The PouThy1 mice maintain normal hearing and balance until being treated with DT (Figure 1A), which ablates all their inner and outer hair cells.^39^ The deafness of the treated mice can be examined by imaging cortical auditory responses through a clear skull (Figures 1B and 1C). In awake mice, we compared the auditory responses to audible broadband noise and visual responses to light flashes among three groups, which were Thy1 mice treated with DT (Thy1-DT), PouThy1 mice treated with saline (PouThy1-saline), and PouThy1 mice treated with DT (PouThy1-DT). Audible sound (90 dB sound pressure level) activated the auditory cortex and other cortical regions in Thy1-DT and PouThy1-saline groups while no such activation pattern was observed in the PouThy1-DT group (Figures 1D–1F), suggesting DT can effectively deafen PouThy1 mice. In contrast, light flashes (80 ms in duration) evoked similar calcium responses in the visual cortex among all the three groups, suggesting that DT does not damage non-auditory cortex (Figure 1G).

### Deafening reduces or eliminates off-target widespread cortical activation to US

**Figure 2:**
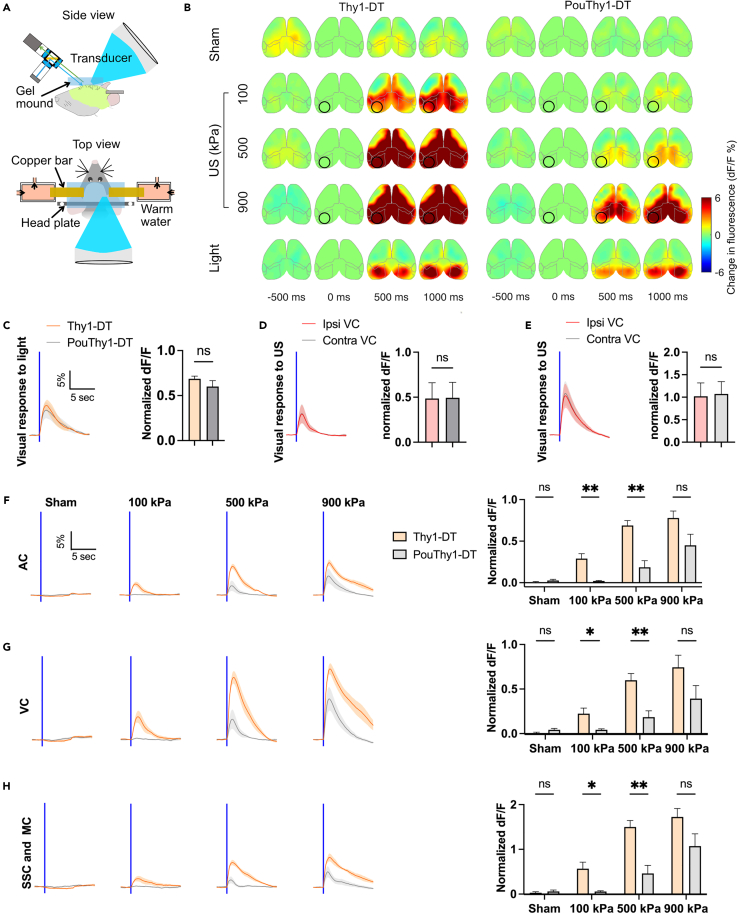
Deafening reduces off-target responses to 270 kHz ultrasound (A) Illustration of simultaneous ultrasonic neuromodulation (UNM) and wide-field cortical imaging. The ultrasound (US) transducer and the imaging equipment were both angled at 45° from parallel to allow optical access to the focus. The transducer was immersed in a cone filled with degassed water. The cone was then coupled to the skull with degassed US gel, which was flattened with a glass plate. The temperature of the gel mound was regulated at approximately 35°C using bilateral copper bars via heat conduction. The other ends of the bars were sealed in 3D printed tubes and submerged in circulating warm water to maintain a constant temperature. (B) Representative examples of cortical activation map from one normal hearing mouse (Thy1-DT) and one deafened mouse (PouDTR-DT) to sham, US (270 kHz center frequency, 500 ms PD, pressure at 100, 500, and 900 kPa), and light flashes. The US target zone is shown as a black circle. The boundary maps are the same as in Figure 1. In the sham trials, no stimulus was presented or applied. (C) Visual responses to light flashes of normal hearing and deafened mice, and the normalized (detailed in STAR Methods) peak dF/F of the two groups (n = 6 mice for each group, unpaired t test, two-tailed, p = 0.2656). The onset time of stimulation is shown as a vertical blue line. (D and E) Cortical responses at ipsilateral (ipsi) US focus and its contralateral (contra) counterpart to US at 500 kPa (D) and 900 kPa (E) and light flashes. The normalized peak dF/F are compared (n = 6 animals, unpaired t test, two-tailed, p = 0.9755 and 0.8999 for D and E, respectively). (F–H) Auditory, visual, somatosensory, and motor responses to sham and US at different pressures. Normalized peak dF/F are compared between the two groups (n = 6 animals for each group, unpaired t test, two-tailed, ∗p < 0.05, ∗∗p < 0.01, ns is not significant). Mean trace is solid and SEM is shaded. Bar graph values represent mean ± SEM.

We further investigated if the PouThy1-DT mouse model could eliminate the auditory confounds and the off-target widespread cortical activation reported by previous UNM studies.^29^^,^^30^ Our *in vivo* UNM setup comprised a wide-field camera and a single element transducer angled to the brain for simultaneous FUS stimulation and calcium imaging.^30^ We used this setup to avoid potentially artifactual mechanical interactions between recording electrodes and FUS, and because wide-field imaging enables us to capture larger areas of the brain, facilitating the assessment of off-target and localized effects caused by FUS. The tip of the transducer (fundamental frequency at 270 kHz and third harmonic at 916 kHz) was manually aligned to the stimulation target, which is −0.5 mm anterior and 2.5 mm lateral of Lambda (Figure 2A). Two previous studies, which primarily examined pulsed parameters in deafened rodents, suggested that these parameters do not cause direct brain activation.^29^^,^^30^ Therefore, in the current study, we primarily focused on a continuous FUS waveform with a pulse duration (PD) of 500 ms, which has been directly demonstrated to effectively activate cultured cortical neurons via specific mechanosensitive calcium channels in an environment free from auditory confounds.^28^ Similar continuous waveforms (PD of 80–640 ms) have been used in various animal and human UNM experiments.^3^^,^^29^^,^^31^^,^^33^^,^^35^^,^^41^^,^^42^^,^^43^^,^^44^ In addition to continuous stimulation, our study examines representative pulsed stimulation parameters.

As expected, when using the 270 kHz FUS, we were able to observe strong auditory confounds and off-target brain activation in normal hearing mice (Thy1-DT). In contrast, in deafened mice (PouThy1-DT), the widespread cortical responses were mostly eliminated when using low peak negative pressure FUS (≤500 kPa). Unexpectedly, we still clearly observed bilateral off-target brain activation from non-auditory regions when increasing the pressure to 900 kPa (Figures 2B and 2F–2H). To examine if this activation was directly elicited by targeted FUS, we compared the ipsilateral and contralateral responses of the visual cortex to FUS, but we did not find that the ipsilateral focus had stronger responses than its contralateral counterpart (Figures 2D and 2E). This suggests that the activation is not likely to be caused directly by local FUS stimulation of the targeted visual cortex.

**Figure 3:**
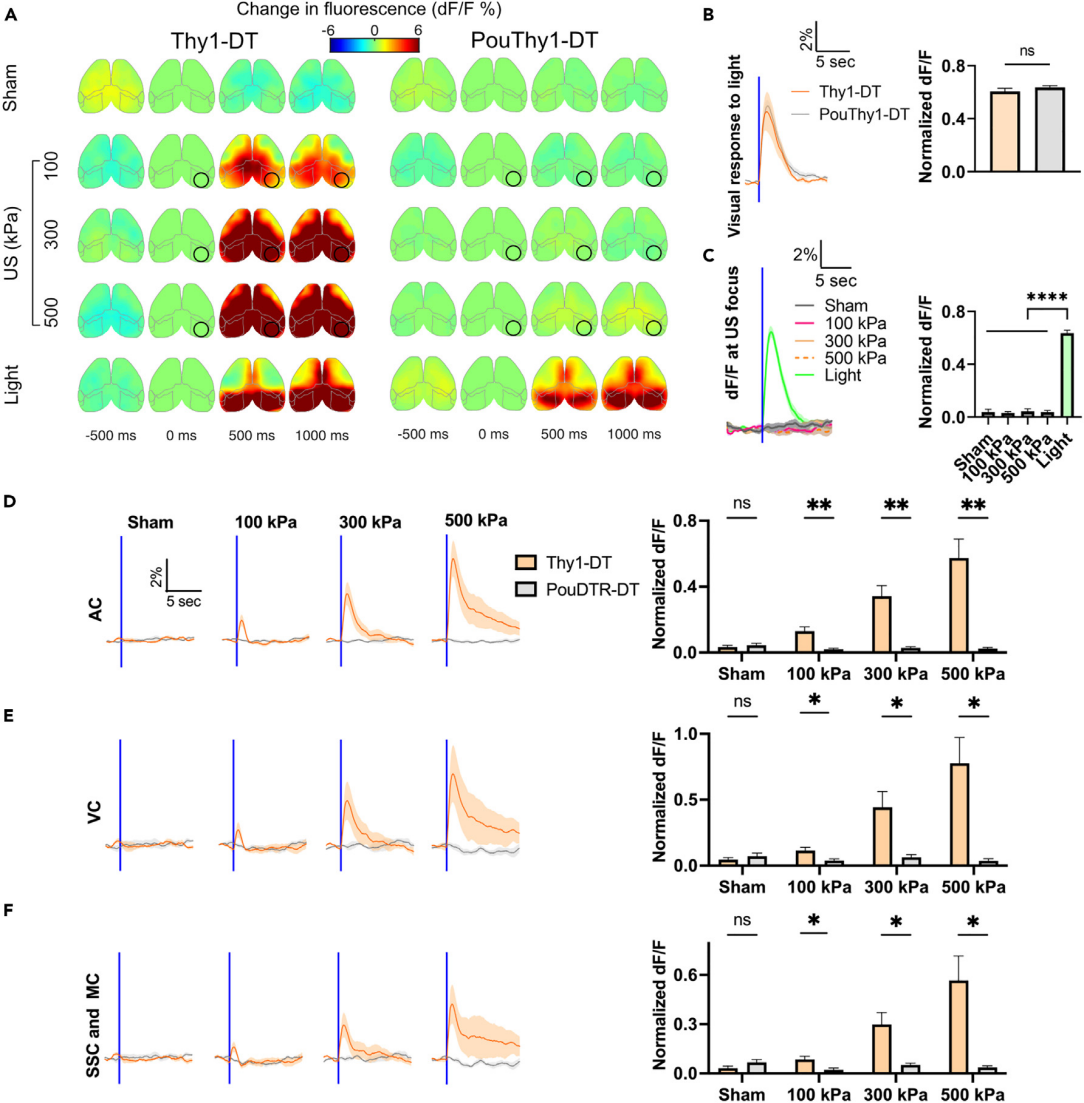
Deafening eliminates off-target responses to 916 kHz ultrasound (A) Representative examples of cortical activation map from one normal hearing mouse (Thy1-DT) and one deafened mouse (PouDTR-DT) to sham, US (916 kHz, 500 ms PD, pressure at 100, 300, and 500 kPa), and light flashes. The US target zone is shown as a black circle. In the sham trials, no stimulus was presented or applied. (B) Visual responses to light flashes of normal hearing and deafened mice, and the normalized peak dF/F of the two groups (n = 6 animals for each group, unpaired t test, two-tailed, p = 0.8618). The onset time of stimulation is shown as a vertical blue line. (C) Cortical responses at US focus to sham, US at different pressures, and light flashes, and the normalized peak dF/F (n = 6 animals, one-way ANOVA ∗∗∗∗p < 0.0001, Tukey’s post comparison) of the deafened mice. (D–F) Auditory, visual, and somatosensory and motor responses to sham and US at different pressures. Normalized peak dF/F are compared between the two groups (n = 6 mice for each group, unpaired t test, two-tailed, ∗p < 0.05, ∗∗p < 0.01, ns is not significant). Mean trace is solid and SEM is shaded. Bar graph values represent mean ± SEM.

To assess the impact of focal zone size, we stimulated with the third harmonic of our transducer, at 916 kHz, with a lateral full width at half maximum (FWHM) pressure profile of 1.4 mm so that the focus can be confined within one hemisphere (Figures 3 and S2). As expected, in normal hearing mice, the application of FUS to the visual cortex resulted in widespread cortical activation at pressure as low as 100 kPa. In contrast, in fully deafened mice, the off-target brain activation was eliminated at pressures up to 900 kPa. Taken together, these results reinforce the importance of using fully deafened mice for UNM experiments because FUS can produce strong auditory and widespread brain activation in normal hearing mice at pressure as low as 100 kPa. In addition, they reveal the additional concern of non-auditory side effects when using low-frequency transducers with large focal zones relative to brain size.

### US reduces focal fluorescence in deafened mice via a thermal mechanism

**Figure 4:**
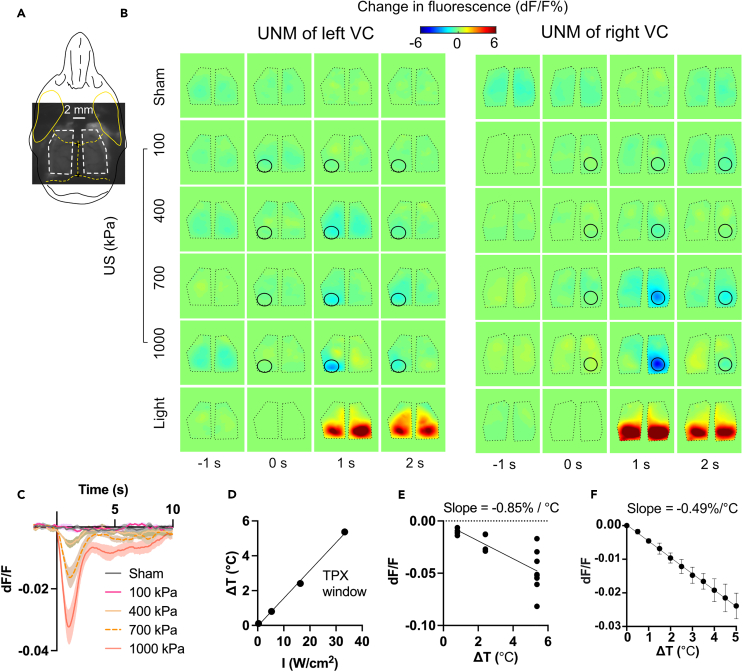
Temperature dependence of fluorescence in *in vivo* deafened mouse brain and *in vitro* human cells (A and B) Two representative examples of cortical activation map in response to sham, US at different pressures (916 kHz, 500 ms PD, pressure at 100, 400, 700, 1,000 kPa), and light flashes. A raw image of a TPX window mouse is shown in A. The length and posterior width of each window are approximately 7 and 4 mm, respectively, allowing for complete acoustic access to the brain for the 916 kHz US. The US target zone is shown as a black circle. The white/black polygonal dots represent the boundaries of the TPX windows. In the sham trials, no stimulus was presented or applied. (C) Focal calcium responses to sham and US at different pressures (i.e., 100, 400, 700, 1,000 kPa) across four animals. Mean trace is solid and SEM is shaded. (D) *In vivo* measurement of the brain temperature increases during UNM. Intensities of 0.33, 5.33, 16.33, 33.33 W/cm^2^ correspond to pressures of 100, 400, 700, 1,000 kPa, respectively. The measured temperature increase linearly correlates with the US intensity (R^2^ = 0.997). (E) Fluorescence change is plotted against measured temperature increase with a slope of −0.85%/°C for *in vivo* brain. (F) Fluorescence change is plotted against measured temperature increase with a slope of −0.49%°C for *in vitro* HEK293T cells.

In the clear skull preparation, we noticed a decrease in local calcium indicator fluorescence following high pressure (900 kPa, 500 ms PD) FUS stimulation (Figure S2), which is in line with the temperature-dependent fluorescence dips reported in another recent UNM study.^42^ To better characterize the relationship between continuous FUS heating and fluorescence without the potentially distorting effects of the skull, we replaced bilateral skull regions with TPX, an acoustically and optically transparent polymethylpentene plastic, 0.125 mm in thickness.^45^ The windows covered +3 to −4 mm anteroposterior (AP), +1 to +5 mm mediolateral (ML) for each hemisphere (Figure 4A). The large windows can accommodate the full 916 kHz FUS focus and allow imaging of major portions of the bilateral motor, somatosensory, and visual cortices.

When increasing the peak positive pressure from 100 kPa to 1,000 kPa in increments of 300 kPa, we observed the decrease of focal fluorescence following the onset of FUS, which then gradually returned to the baseline (Figures 4B and 4C). We measured the temperature change at the brain surface under the TPX window with a miniaturized thermistor during UNM in separate animals (Figures 4D and S5) and calculated our *in vivo* preparation having a temperature dependence of fluorescence of approximately −0.85%/°C (Figure 4E), relatively consistent with Estrada’s result of −1.9 ± 0.7%/°C in brain slices.^42^ The difference may be due to heating of the TPX window material or the thermistor used for our measurement, leading to an overestimation of temperature changes. Further investigations should account for factors like the skull and/or cranial window during thermal modeling,^11^^,^^46^^,^^47^ and use miniaturized devices for temperature measurement.

To study the effects of temperature on cellular GCaMP fluorescence, we heated a population of GCaMP6f-expressing human HEK293 cells using a qPCR machine from 37°C to 42°C in 1-degree increments and simultaneously measured cellular fluorescence. We found a linear decrease in fluorescence intensity when the temperature increased (Figure 4F), suggesting that the observed US-induced fluorescence dimming *in vivo* could, at least in part, be attributed to the US-induced elevation in temperature.

### High-pressure US elicits focal, spreading depolarization with underlying tissue damage

**Figure 5:**
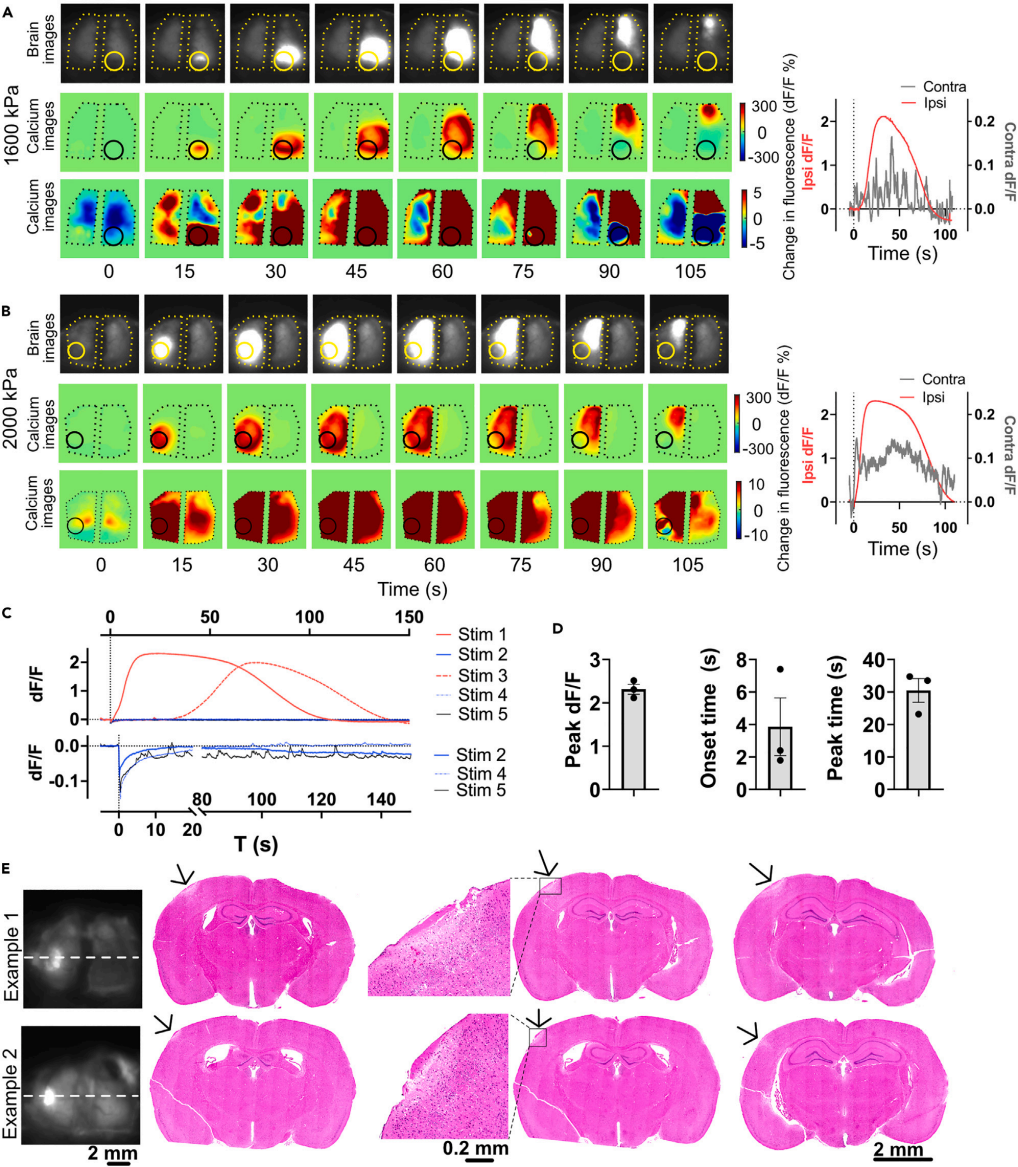
Ultrasound produces localized and hemispherically spreading disruptive depolarization (A and B) Two representative examples of cortical activation maps of localized depolarization by US in deafened animals. The depolarization started at the focus and then propagated over the ipsilateral hemisphere. The right panels are the dF/F of the ipsilateral focus (red curve) and its contralateral counterpart (black curve). The ipsilateral dF/F is approximately 20-folds of the contralateral dF/F. The US target zone is shown as a yellow/black circle. The black polygonal dots represent the boundaries of the TPX windows. The onset time of stimulation is shown as a vertical dot line in the right plots. (C) The dF/F at the focus in response to five consecutive US stimulation (2,000 kPa, 500 ms PD) with an interval of 10 min. The zoomed curves are shown below. Only trial #1 and #3 elicited depolarization. (D) The peak dF/F is 2.31 ± 0.20, onset time is 3.87 ± 3.07, and peak time is 30.5 ± 6.35 s, respectively (n = 3 animals). Bar graph values represent mean ± SEM. (E) Examples of H&E staining at the sonication site suggest that high-pressure US (2,000 kPa, 500 ms PD) induced brain damage (white regions with low cell density, as pointed by black arrows) at the focus.

Having failed to obtain evidence of direct brain activation in awake deafened mice with FUS at pressures up to 1,300 kPa and a long interstimulus interval (ISI) of 155 s (Figure S3), we further tested if pressures of 1,600 kPa and above (in increments of 400 kPa at 916 kHz) could elicit stronger calcium responses, potentially overcoming the negative fluorescence dip caused by heating. At this higher pressure, we observed a very strong calcium signal at the focus (∼200% peak dF/F, Figure 5), which was ∼20-fold larger than that elicited by sensory stimuli (e.g., Figure 1). Originating at the FUS focus, this excitation propagated throughout the ipsilateral hemisphere over approximately 1 min (Figure 5). This spatiotemporal pattern is similar to that elicited by focal cortical seizures.^48^^,^^49^ On the contralateral side, there was a relatively weaker (about 15% peak dF/F) brain activation having similar duration with the ipsilateral response. We also found that the strong focal calcium signal was not readily repeatable in the same animal. As shown in an example (Figure 5C), when performing five consecutive FUS trials (Stim 1 to 5) at an interval of 10 min in one experiment, only the first and third trials resulted in signal, indicating potential tissue damage. To determine effects on the tissue, we perfused three animals 24 h after they received a single pulse of US stimulation (2,000 kPa, 500 ms PD, 916 kHz) and performed H&E staining, with two animals showing clear damage in the area of stimulation (Figure 5E). The combined live imaging and histological evidence suggests that the strong propagating calcium signal was due tofocal neuronal damage leading to spreading activation over the ipsilateral cortex, rather than direct non-damaging US stimulation.

### Low-pressure pulsed US does not elicit localized calcium signals

**Figure 6:**
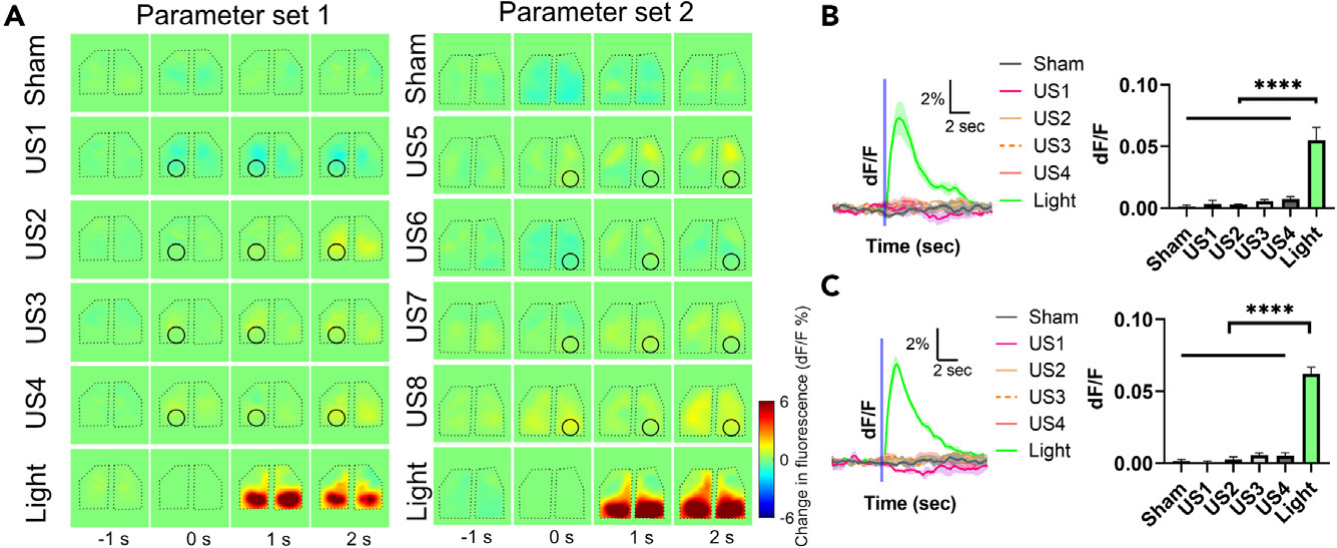
Cortical responses to low-pressure pulsed ultrasound (A) Two representative cortical activation maps at different time points in response to sham, US (parameter set 1 and 2), and light flashes to both eyes. The US target zone is shown as a black circle. In the sham trials, no stimulus was presented or applied. The black polygonal dots represent the boundaries of the TPX windows. (B and C) Responses of the targeted region of visual cortex to sham, different US parameters (parameter set 1 [B] and parameter set 2 [C]), and light flashes (n = 4 animals, one-way ANOVA ∗∗∗∗p < 0.0001, Tukey’s post comparison). The onset time of stimulation is shown as a vertical blue line. Mean trace is solid and SEM is shaded. Bar graph values represent mean ± SEM.

After comprehensively characterizing responses to continuous FUS, we also tested pulsed stimulation parameters (Table 1) informed by UNM studies that have reported that low-intensity pulsed FUS can elicit electrophysiological activity at the FUS focus.^2^^,^^50^ To keep the FUS focus on the ipsilateral target and minimize somatosensory effects, we used 916 kHz FUS instead of 250–500 kHz FUS used in their studies. We did not observe any activation in our deafened mice with TPX window (Figure 6), even with 3- to 4-fold higher pressure than used in previous reports. In contrast, we observed strong bilateral brain activation in normal hearing mice using the same parameters (Figure S4), suggesting that auditory confounds may contribute to the widespread brain activation elicited by pulsed FUS.

**Table 1:** Pulsed ultrasound parameters investigated in deafened mice

Parameter		f (MHz)	c/p	PD (ms)	PRF (Hz)	Duty cycle	Np	US Stim length (ms)	Pr (kPa)	ISPPA (mW/cm2)	ISPTA (mW/cm2)
Set1	US1	0.916	210	0.229	2500	57.3%	150	60	100	330	189
	US2	0.916	210	0.229	2500	57.3%	150	60	300	3000	1719
	US3	0.916	183	0.2	1500	30%	120	80	100	330	99
	US4	0.916	183	0.2	1500	30%	120	80	300	3000	900
Set2	US5	0.916	18	20	30	60%	2	67	72.6	175.80	105.48
	US6	0.916	18	20	30	60%	2	67	300	3000	1800
	US7	0.916	2	2	300	60%	20	67	72.6	175.80	105.48
	US8	0.916	2	2	300	60%	20	67	300	3000	1800

The pulse patterns of parameter set #1 overlapped with that being used in Tufail’s study^2^ while the pulse patterns of parameter set #2 overlapped with that being used in Yu’s study.^50^ These two studies are representative of many others in the field.^19^ Note that we used 916 kHz FUS with smaller focus to minimize the off-target effects induced using a low-center frequency transducer.

## Discussion

This study presents a new conditionally deafened mouse model for studying the effects of FUS on neural activity *in vivo* and detailed characterization of the cortex-wide calcium dynamics of deafened mice in response to continuous FUS with 500 ms PD, at pressures ranging from 100 kPa to 2,400 kPa. In clear skull preparation, by eliminating the auditory confounds, we observed a significant reduction in off-target brain activation with 270 kHz FUS at pressures up to 500 kPa, and complete elimination of off-target activation with 916 kHz FUS at pressures up to 900 kPa. However, we did observe residual brain activity for the 270 kHz frequency at high pressures of 500 and 900 kPa, which may be attributed to direct activation of somatosensory organs due to the wide beam of the 270 kHz FUS (∼8.85 mm lateral FWHM for pressure) as these receptors are excitable to FUS stimulation.^43^^,^^51^^,^^52^^,^^53^

Using the TPX window preparation with 916 kHz continuous FUS for 500 ms PD, we observed pressure-dependent off-target and localized effects. After eliminating the auditory confounds, we observed localized temperature-dependent fluorescence dimming starting from pressure at 400 kPa due to fluorophore heating (Figure 4), which is consistent with a previous report.^42^ When we increased the pressure to 1,000 and 1,300 kPa, we occasionally observed bilateral off-target neural activation at the visual and somatosensory cortices in conjunction with dimming of ipsilateral fluorescence (Figure S3). When we further increased the pressure to 1,600 kPa or above, we observed strong focal depolarization with spreading to ipsilateral cortex, accompanied by damage to brain tissue and contralateral off-target sensory activation.

**Figure 7:**
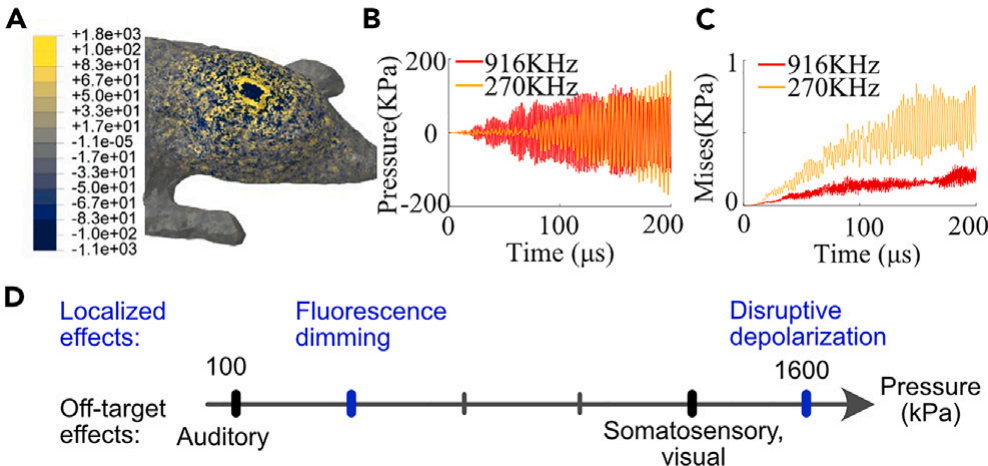
Localized and non-localized effects and potential mechanisms of *in vivo* UNM in mice (A) Result of finite element method (FEM) model of a mouse, where an FUS with peak amplitude of 1,000 kPa and frequency of 916 kHz is applied as a normal pressure introduced as a Neumann boundary condition. The results depict the pressure contours after 200 μs of explicit dynamics simulations. Note the pressure is widely distributed on the head. (B and C) Time history of off-target pressures and von Mises stress at a region on the face, obtained from two separate FEM simulations with identical FUS peak amplitudes of 1,000 kPa but distinct frequencies. (D) US can elicit auditory confounds at low pressure. In deafened mice, the auditory confounds can be greatly reduced or eliminated. However, the thermal confounds at the focus, other sensory confounds, and destructive depolarization will be observed as the pressure of the US increases.

To understand the off-target residual sensory effects in deafened mice induced by FUS, we created a finite element model (FEM) of a mouse^54^ and used it to investigate the response of mouse models to transcranial FUS applied to the visual cortex (Figure 7). We found that the induced wave pattern is complex and has both localized and delocalized components, suggesting that peripheral skin receptors and photoreceptors may experience displacements and stresses at levels that could result in the activation of ascending somatosensory and visual pathways, as reported in some peripheral UNM studies.^43^^,^^52^^,^^53^^,^^55^ We also computed the off-target stresses at a small region on the mouse neck, where we observed that the shear stresses (i.e., von Mises stresses), unlike pressures, exhibit a strong frequency dependence in their response (Figures 7B and 7C). These modeling results, together with our experimental data, establish a range of pressure-dependent localized and off-target effects of FUS UNM (Figure 7D). Furthermore, as auditory confounds have been reported both with perpendicular and angled transducers,^29^^,^^30^^,^^35^^,^^50^ future studies should investigate the detailed differences in sound and shear wave propagation patterns in the mouse skull and their implications for UNM.

We found no evidence that FUS evokes direct calcium responses in the live brain (Figures 3, 4, and S3) at parameters that were able to do so in cultured neurons (Figure S6).^28^ This discrepancy may be due to biophysical differences, as the brain may experience different mechanical and acoustic conditions than cultured neurons. Moreover, the expression levels of the US-sensitive and amplifier channels identified in cultured embryonic neurons (TRPP1/2, TRPC1, and TRPM4) may be different in the adult mouse brain. Future studies may explore cell-type responses to FUS in fully deafened animals to better understand this discrepancy.

Previous studies investigated the correlation between FUS parameters and motor readouts of brain activation (i.e., electromyography [EMG] signals) in normal hearing, lightly anesthetized animals.^3^^,^^4^^,^^41^ In our study, we used fully deafened, awake mice and relied on cortical calcium responses as direct indicators of brain activation. Due to these differences, the findings concerning effective parameters in previous studies may not directly map onto ours.

However, our results do not conclusively demonstrate that FUS is *unable* to produce direct brain activation. For example, although a comprehensive set of continuous FUS parameters had been tested, including 270 kHz and 916 kHz center frequency, other frequencies and PDs could yield different results. Furthermore, moderate-pressure FUS may have generated weak activation masked by temperature-dependent fluorescence reduction. Similarly, high-pressure FUS may have produced brain activation that was overwhelmed by the intense seizure-like depolarization attributed to cellular damage. Finally, there might be subcortical activation and finer cortical activation that cannot be observed by wide-field calcium imaging.^30^^,^^56^ Therefore, other neural recording approaches, such as functional US imaging or two-photon microscopy, may be needed in future UNM studies to record FUS-elicited neural activity in deep brain or at single-neuron resolution.^2^^,^^19^^,^^57^^,^^58^^,^^59^

Our study employed a conditionally deafened animal model, which exhibits nearly complete hair cell loss in the cochleae and utricles,^39^ making it an optimal model for studying the use of treatments such as deep brain stimulation, transcranial magnetic stimulation, and transcranial direct current stimulation, for hearing and vestibular disorders. More immediately, this model could be useful for sonogenetics studies, which have reported localized brain activation with FUS by expressing or overexpressing various mechanosensitive and/or thermosensitive ion channels (MscL, Prestin, TRPA1, TRPV1) in the brain of normal hearing mice.^44^^,^^57^^,^^58^^,^^59^^,^^60^ However, it is crucial to use models like our conditionally deafened mice because our previous and current experimental and modeling results have shown that transducers at center frequencies lower or around 1 MHz could elicit strong auditory confounds and off-target brain activation.^29^^,^^30^^,^^54^ Moreover, researchers should also pay attention to somatosensory and visual confounds, as high-pressure FUS may still activate ascending non-auditory pathways in deafened animals. To distinguish brain activation from disruptive depolarization, it is necessary to perform histology and confirm that the FUS parameters leading to brain activation do not cause any brain damage. In conclusion, UNM holds great potential in treating various brain disorders,^61^^,^^62^ and it is always advisable to stay cautious by imaging whole cortical or brain activity and performing control experiments to ensure that the observed brain activation is localized and not confounded. Further studies using optical readouts in conditionally deafened animal models will help validate parameters and mechanisms for translational UNM and sonogenetics in humans and help define a parameter range considered safe from thermal and other potentially damaging effects.

### Limitations of the study

This study explored a limited range of cortically targeted FUS parameters using wide-field calcium imaging in awake, deafened Pou4f3^+/DTR^ × Thy1-GCaMP6s mice. While we did not observe direct neural stimulation, we do not rule out that such stimulation may be possible and observable with alternative FUS parameters, neural recording methods (e.g., functional US imaging, two-photon microscopy, electrophysiology), and target brain region.

## STAR★Methods

### Key resources table

**Table:** 

REAGENT or RESOURCE	SOURCE	IDENTIFIER
**Bacterial and virus strains**		

AAV1 viral vector GCaMP6f	Addgene	100837-AAV1; 100837-Vdna
Biological samples		
Diphtheria toxin	Sigma-Aldrich	D0564-1MG

**Chemicals, peptides, and recombinant proteins**		

Isoflurane	Henry Schein	IsoThesia, SKU 029405
Ophthalmic Ointment	Dechra Veterinary Products	Puralube
Super Glue	BAZIC Products	#2007
High-calorie gel	Nutri-Cal	Tomlyn/Vétoquinol
C&B METABOND Quick Base	Supply Clinic	S398
C&B-METABOND L-Powder Clear	Supply Clinic	S399
"C" Universal TBB Catalyst	Parkell	SKU:S371
Poly-d-lysine	Millipore Sigma	P1274
GlutaMax	Gibco	35050061
B27	Thermo Fisher Scientific	17504044
Glutamate	Millipore Sigma	G1251
Penicillin/streptomycin	Corning	30-002-CI
Polymethylpentene (TPX)	Sigma-Aldrich	GF69297687

**Deposited data**		

Raw and analyzed data	This paper	Open Science Framework: https://osf.io/4qsed/

**Experimental models: Cell lines**		

HEK293T	ATCC	CRL-3216; RRID:CVCL_0063
Neuron cultures	Yoo et al.^28^	N/A

**Experimental models: Organisms/strains**		

Tg(Thy1-GCaMP6s)GP4.12Dkim	The Jackson Laboratory	RRID: IMSR_JAX:025776
B6.Cg-Pou4f3^tm1.1(HBEGF)Jsto^/RubelJ	The Jackson Laboratory	RRID:IMSR_JAX:028673
C57BL/6J	The Jackson Laboratory	RRID: IMSR_JAX:000664

**Software and algorithms**		

LabVIEW 2022	National Instruments	RRID:SCR_014325
MATLAB 2022a	MathWorks	RRID: SCR_001622
GraphPad Prism 9	GraphPad Software	RRID:SCR_002798
Experiment control software	This paper	Open Science Framework: https://osf.io/4qsed/

**Other**		

Ultrasound transducer	Sonic Concepts	SN-115
Signal generator	Keysight Technologies	33522B
RF amplifier	Electronics & Innovation	1020L
Multi-Field Magnetic Speakers	Tucker-Davis Technologies	MF1
Stereo Power Amplifier	Tucker-Davis Technologies	SA1
PXIe chassis	National Instruments	PXIe-1073
Function generator	National Instruments	PXI-5421
Oscilloscope	Keysight Technologies	DSO-X 2004A
Fiber optic hydrophone system	Precision Acoustics	FOH
Fiber optic hydrophone	Precision Acoustics	PFS and TFS
Objective lens	Thorlabs	AC254-060-A
Tube lens	Thorlabs	AC254-040-A
Fluorescence filter set	Thorlabs	MDF-GFP
Camera	PCO	pco.panda 4.2
THERMISTOR NTC 10KOHM 3380K BEAD	Murata Electronics	NXFT15XH103FA2B025

### Resource availability

#### Lead contact

Further information and requests should be directed to the Lead Contact, Dr. Mikhail Shapiro (mikhail@caltech.edu).

#### Material availability

This study did not generate new unique reagents.

#### Data and code availability

• Data have been deposited on the Open Science Framework (Open Science Framework: https://osf.io/4qsed/) and are publicly available as of the date of publication.
• Original code has been deposited on the Open Science Framework (Open Science Framework: https://osf.io/4qsed/) and is publicly available as of the date of publication.
• Any additional information required to reanalyze the data reported in this work is available from the lead contact upon reasonable request.

### Experimental model and study participant Details

#### Animals

All animals were used in accordance with animal procedures approved by the Institutional Animal Care and Use Committee and the Institutional Biosafety Committee at the California Institute of Technology. For control experiments, transgenic mice, C57BL/6J-Tg(Thy1-GCaMP6s)GP4.12Dkim/J (The Jackson Laboratory, Stock No. 025776) were used. Strain B6.Cg-Pou4f3tm1.1(HBEGF)Jsto/RubelJ (The Jackson Laboratory, Stock No. 028673) and C57BL/6J-Tg(Thy1-GCaMP6s)GP4.12Dkim/J were crossed to breed the double transgenic heterozygous Pou4f3^+/DTR^ x hemizygous Thy1-GCaMP6s mice, which were used for the deafening procedures. Experiments used health male and female adult mice (20-35 g) older than eight weeks of age. Animals were group-housed at a maximum of five per cage by gender with *ad libitum* access to food and water and were maintained on a 12/12 h normal light-dark cycle.

#### Primary cell cultures

The detailed method of primary neuron preparation has been described in the previous study from our laboratory.^28^ The cortical neurons collected from embryonic day 18 C57BL/6J mice (The Jackson Laboratory) were seeded on the top of poly-d-lysine (Millipore Sigma, 70-150k mol wt) pre-coated Mylar dish at a density of 300 cells/mm^2^ , and maintained in Neurobasal medium (Thermo Fisher Scientific) supplemented with B27 (2% v/v, Thermo Fisher Scientific), GlutaMax (2 mM, Gibco), glutamate (12.5 μM, Sigma) and penicillin/streptomycin (1% v/v, Corning) in a humidified incubator with 5% CO2 and 37°C. Half of the medium was changed with the fresh medium without glutamate every 3 days, and neurons were used for ultrasound stimulation experiments after 11–12 days from the seeding. For the experiments characterizing the temperature dependence of fluorescence *in vitro*, a HEK293 cell line constitutively expressing GCaMP6f under the CMV promoter were cultured in Dulbecco’s modified Eagle’s medium (DMEM) supplemented with 10% tetracycline-free fetal bovine serum (FBS) and penicillin/streptomycin.

### Method details

#### Animal surgery

Anesthesia was induced by placing mice in a clean induction chamber and delivering 5% isoflurane. The animal’s hair was removed and was then placed in a stereotax. The head was held steady using ear bars and a nose cone. Anesthesia was maintained via delivery of isoflurane (1.5%-2%) in the nose cone. The body temperature was maintained using a heating pad. Both eyes were protected using ophthalamic ointment. The residual hair was removed using hair removal cream and exposed scalp sterilized using chlorhexidine. Local anesthetic bupivacaine 0.5% was injected just under the scalp and left for 1 minute before any incisions. The skull was then exposed via an incision along the midline and laterally above the cerebellum.^30^ For clear skull preparation, a thin layer of super glue was evenly applied to the exposed skull.

To avoid the aberrance from the skull, in some experiments, we replaced bilateral partial skull with an acoustically and optically transparent polymethylpentene plastic (TPX, Mitsui Chemicals, Minato City, Japan), 0.125 mm in thickness. For TPX implantation surgery, the animals were given subcutaneous injections of an osmotic diuretic Manitol 20% (≤ 0.5ml) to prevent brain swelling. The size of the TPX window on each side is approximately 7 mm × 4 mm (-4 to +3 mm along the antero-posterior axis; +1 to +5 mm from the midline along the lateral axis) to expose major motor, somatosensory and visual cortices. To prevent heating of cortex, the drilling of the craniotomy was done slowly, sterile saline rinse between short periods of drilling. Angled forceps were used to remove the skull pieces carefully. Next, a pre-cut TPX window was positioned on top of the exposed brain and sealed with tissue adhesive (Vetbond, veterinary grade, 3M).

#### Administration of diphtheria toxin

Diphtheria toxin was used in accordance with the procedures approved by the Institutional Biosafety Committee at the California Institute of Technology. We used the same protocol described in Golub’s study.^39^ Adult mice received two intramuscular injections of diphtheria toxin (Sigma-Aldrich D0564-1MG) at 50 ng/g, spaced 2 days apart. Mice received 0.4 ml of lactated Ringer's solution by subcutaneous injection once or twice daily on days 3–6 after the first DT injection. Between days 1 and 6 after the first DT injection, moist food was provided and was supplemented with high-calorie gel (Tomlyn/Vétoquinol from Nutri-Cal).

#### *In vivo* experimental preparation

Each experiment day, anesthesia was induced by placing mice in a clean induction chamber and delivering 5% isoflurane. As soon as voluntary movement ceased, mice were quickly moved to the UNM setup and maintained at 1-2% isoflurane for preparation. The headplate of the animal was fixed to two aluminum bars. The body of the animal was restrained to a platinum plate with a piece of tape. The skull was thoroughly rinsed and cleaned with sterile saline. The ultrasound transducer was immersed into a 3-D printed cone filled with degassed water. To target the transducer to the specific brain region, we initially affixed a 3-D printed cone tip to the ultrasound cone. The focal point of the ultrasound was set to be 1 mm below the tip. We then utilized a manual 3-axis micrometer (XYZ) stages to move and advance the cone, angled 45 degrees from parallel, to the approximate target (i.e., left or right visual cortex). The cone tip gently touched the skull or TPX window. Subsequently, we removed the cone tip and filled the resulting gap with degassed ultrasound gel, which was then flattened with a glass plate for optical access. In the case of TPX window mice, we additionally validated the focus of the FUS by visualizing it through thermal fluorescence dimming. Before each experiment, the degassed coupling ultrasound gel was preheated in a water bath to 37°C. Subsequently, the gel was quickly applied to the surface of the skull or TPX window to establish coupling between the brain and the ultrasound cone. Throughout the entire experiment, the temperature of the gel was maintained between 35°C and 38°C by using bilateral copper bars (Figure 2A), with each bar's other end sealed in a 3D printed tube filled with circulating warm water from a water bath. The gel temperature at the target site was monitored every 10-20 minutes using a thermocouple that was carefully inserted into the gel above the brain target. After each measurement, the thermocouple was slowly pulled out to avoid potential electrical signal interface to UNM experiments. To measure the temperature change at the brain surface due to FUS stimulation (Figure S5), we implanted a miniaturized (1 × 0.5 mm in dimensions) thermistor (NXFT15XH103FA2B025, Murata Electronics, Japan) under the TPX window and acquired the temperature data with an Arduino connected to the thermistor. Before acquiring images, the isoflurane was turned off and the mouth cone was retracted to allow the mice breathing fresh air. Experiments started after the animals were fully awake and had voluntary movements.

#### Experimental protocol design

All imaging animals underwent 50 blocks of experiments. In each block, a trial of each stimulus was presented once in random order. The ISI was 20 s for all experiments except 155 s in Figure S3. In Figure 1, the stimuli in each block were sham, light flash to both eyes, and audible broadband noise to both ears. In Figures 2, 3, 4, and 6, the stimuli in each block were sham, ultrasound at different pressures, and light flash to both eyes. In Figure 5, due to the long ISI, each block included only one stimulus, which could be either sham, US at 1000 kPa, or US at 1300 kPa. In Figure 5, only one or five trials were presented. In Figures 2 and S4B, the transducer was operated at its fundamental frequency of 270 kHz, while in all other experiments it was operated at 916 kHz. Continuous ultrasound with a PD of 500 ms was utilized for all experiments, except for Figures 6 and S4B, in which we examined some pulsed ultrasound parameters.

#### Experimental control

Experiments were controlled by custom software, written in LabVIEW (National Instruments, Austin, TX) and MATLAB (Mathworks, Natick, MA). A PXIe chassis (PXIe-1073) and a function generator (PXI-5421), both from National Instruments, were used to generate a ramped broadband noise waveform which was amplified by a power amplifier (SA1, Tucker-Davis Technologies, Alachua, FL) to drive an open-field magnetic speaker (MF1, Tucker-Davis Technologies, Alachua, FL). The speaker-ear system was calibrated using a condenser microphone system (PS9200KIT, ACO Pacific, Belmont, CA).^29^ The light flashes were generated by two white LEDs driven by an Arduino UNO. Another Arduino (Mega 2560) was used to trigger the function generator of audible sounds, Arduino UNO of LEDs, wide-field camera, and the signal generator of ultrasound. This Arduino communicated with MATLAB in a PC, which randomized the stimuli in each block, to trigger the appropriate stimulus (sham, sound, light, or ultrasound) accordingly.

#### Imaging data acquisition

The construction of the wide-field calcium imaging system has been described in the previous study.^30^ In the present study, images were collected at 20 Hz using a camera (pco.panda 4.2, PCO, Kelheim, Germany) running on the external trigger mode from an Arduino. The exposure of each image was set to 39 ms. One hundred frames (i.e., 5 second recording) were acquired before the on-set of stimuli, where the first and last frames were abandoned when calculating the baseline fluorescence F0 to avoid shutter artifact and stimulus onset noise. After that, 200 or 3000 frames (i.e., 10 or 150 s recording) were acquired for recording evoked calcium responses to different types of stimuli.

#### *In vitro* experimental preparation

Water bath temperature of neurons was maintained to 37°C during the US stimulation experiment. For calcium imaging, Syn-driven GCaMP6f as a calcium sensor was delivered to neurons via AAV1 viral vector transfection (Addgene 100837-AAV1, 1E10 vp/ dish) at 4 days *in vitro*. For the experiments characterizing the temperature dependence of fluorescence *in vitro*, cells were trypsinized, resuspended in Opti-MEM (Thermo Fisher Scientific), and added to 200μL qPCR tubes. The qPCR machine was programmed to perform a temperature ramp from 37°C to 42°C in 1-degree increments, while the GCaMP fluorescence was read-out simultaneously using the corresponding FAM fluorescence filters.

#### Ultrasound generation and calibration

Details of ultrasound generation and calibration are described in our previous study.^29^^,^^30^ A single element transducer (H-115, Sonic Concepts, Bothell, WA) was used in all experiments. The transducer can run at its first harmonic (270 kHz) and third harmonic (916 kHz) with different matching boxes, which were driven by a 200 W RF amplifier (E&I 2200, Electronics & Innovation, Rochester, NY). The amplifier was triggered by a waveform generator (33500B series, Keysight Technologies, Santa Rosa, CA).^29^ Calibration was done in a large water tank with a fiber-optic hydrophone system (FOH, Precision Acoustics, Dorchester, UK). Unless stated otherwise, pressures listed in the manuscript refer to un-derated peak negative pressure measured in a water tank.

### Quantification and statistical analysis

All raw data was processed using custom code written in MATLAB. Statistical analysis was performed using the program GraphPad Prism 9 (GraphPad Software, San Diego, CA). All the figures were plotted using MATLAB and Prism. Maximum dF/F signal was used for group comparisons.

#### Imaging data analysis

Each image frame was spatially filtered with a 20 pixel (∼500 μm) square filter to reduce noise. Relative fluorescence intensity changes were calculated as dF/F = (F- F0)/ F0. The dF/F peaks at the ROIs (Figures 2 and 3) were normalized to the strongest cortical fluorescence response to light flashes for each animal. For clear skull experiments (Figures 1, 2, 3, S1, and S2), wide-field images were transformed to Common Coordinate Framework atlas coordinates (CCF, v3 ©2015 Allen Institute for Brain Science) by affine transformation based on manually selected control points.^63^ Cortical areas such as visual, auditory, somatosensory and motor cortices were directly used as ROIs. CCF remapping was not performed for the TPX window experiments (Figures 4, 5, 6, S3, and S4) as the control points were not visible. Hence, the ROI for FUS focus was manually selected based on the negative fluorescence region where FUS focal heating occurred.

#### Finite element modeling

We adopt the geometry for our three dimensional finite element model from the co-registered x-ray CT of a male mouse developed in the Digimouse project.^54^^,^^64^ This model contains all the major anatomical parts of the mouse body: skeleton, whole brain, heart, lungs, liver, stomach, spleen, pancreas, kidneys, testes, bladder, muscles and skin. We have discretized the mouse anatomy into a finite element model with more than 10 million three-dimensional linear tetrahedral elements. We have also adopted organ-dependent material properties including elasticity and viscoelasticity. We subject a region on the skin on top of the skull to harmonic pressure with intensity of 1 MPa. In order to have the US pressure focused inside the brain, the pressure is imposed in a phased array manner, where the phase lag is introduced in a radial direction. We have also ensured the spatial and temporal discretization are respectively smaller than ∼1/15 of the wavelength size and satisfy the CFL stability criterion.^54^^,^^65^ We then solved the initial boundary-value problem of small-strain elasticity with recourse to the explicit finite element method (Abaqus/Explicit, Dassault Systèmes Simulia, France).
