# Supplementary material for: Effects of focused ultrasound in a “clean” mouse model of ultrasonic neuromodulation
Source: iScience. 2023 Oct 31;26(12):108372. doi: 10.1016/j.isci.2023.108372 (PMC10690554; doi:10.1016/j.isci.2023.108372)

**iScience, Volume *26***

## Supplemental information

# Effects of focused ultrasound in a “clean” mouse model of ultrasonic neuromodulation

**Hongsun Guo, Hossein Salahshoor, Di Wu, Sangjin Yoo, Tomokazu Sato, Doris Y. Tsao, and Mikhail G. Shapiro**

**Figure S1. Transducer calibration and brain remapping using Allen Mouse Brain Common Coordinate Framework 3.0 (Related to Figures 1-3, and S2).** (A) Calibration of the transducer (Sonic Concepts, model H-115) at its fundamental frequency 270 kHz and the third harmonic 916 kHz, which have a lateral full width at half maximum [FWHM] for pressure of 8.85 mm and 1.4 mm, respectively. (B) The brain remapping used six control points (I) in the top view of Allen Mouse Brain CCF and another six control points from the raw wide-field image and the calcium activity map (II and III), where the points can be manually adjusted. After remapping (IV-V), different cortical regions can be identified and the regional responses (visual, auditory, somatosensory and motor) can be calculated using the remapped ROIs.

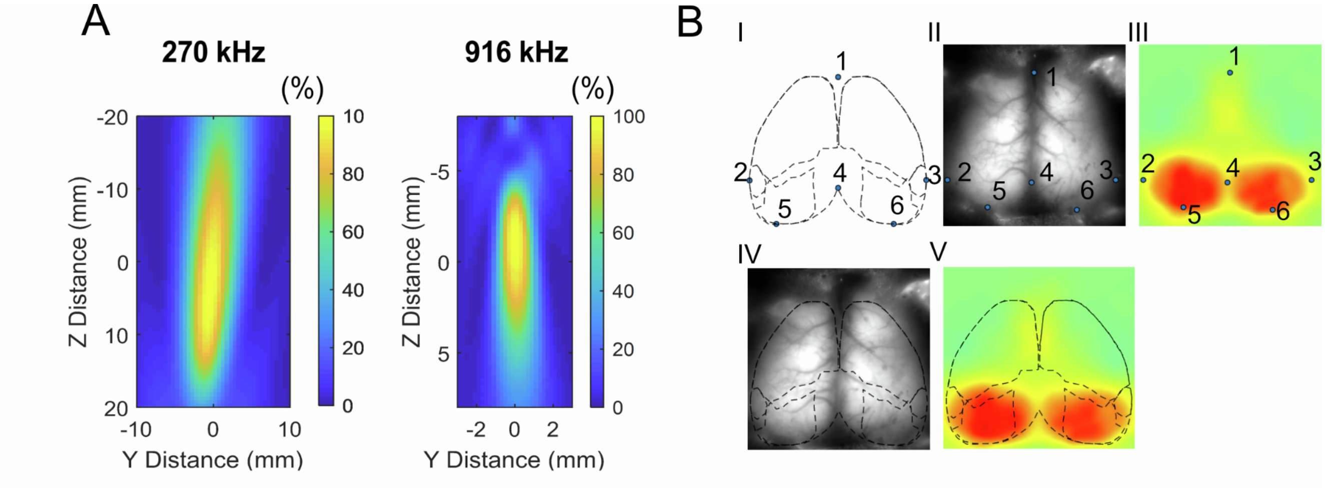


**Figure S2. Ultrasound reduces GCaMP fluorescence at the focus in deafened mice with clear skull preparation (Related to Figure 3).** (A) A representative example of cortical activation map at different time points in responses to sham, ultrasound (500, 700, 900 kPa; 500 ms PD), and light flashes to both eyes. The ultrasound was delivered through the skull in the clear skull preparation. (B) Responses of the focus to different ultrasound parameters and sham (n=4 animals). Clear negative and long-lasting fluorescence signals can be observed for pressures of 700 kPa and 900 kPa. The US target zone is shown as a black circle.

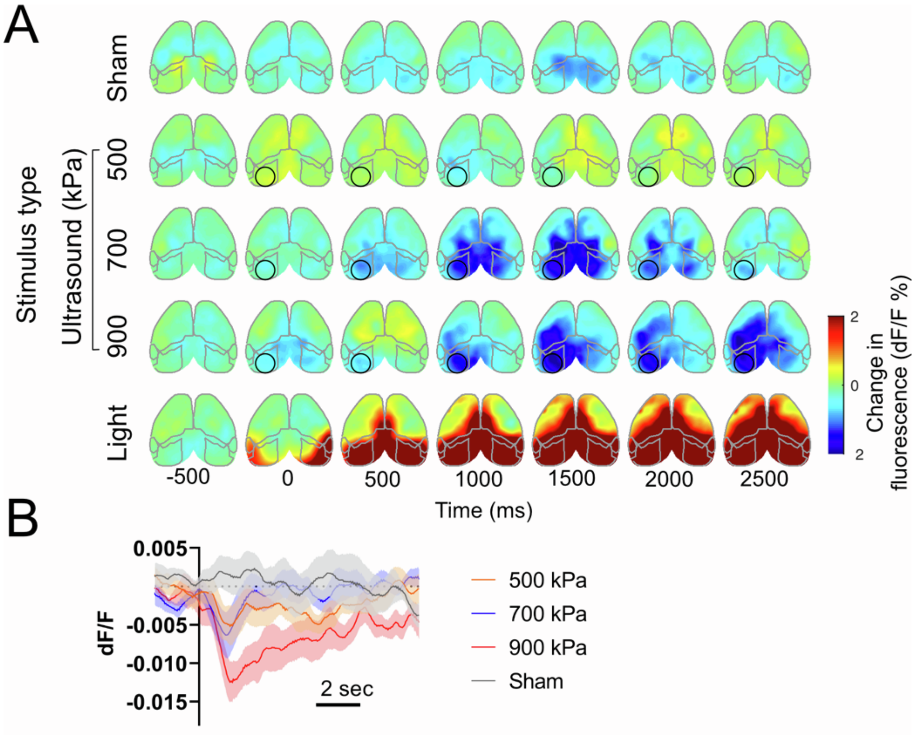

**Figure S3. Off-target cortical responses to ultrasound in deafened mice at higher pressure (Related to Figure 4).**

(A-B) Representative examples of cortical activation map in response to US (916 kHz, 500 ms PD) at 1000 kPa (A) and 1300 kPa (B), respectively. For each pressure, one example (with black arrows) shows bilateral off-target brain activation at the visual and somatosensory cortices in conjunction with localized decrease of fluorescence signal (one out of four animals for each group) while the other one shows only the decrease of the fluorescence signal at the focus. A complex cortical activation pattern, including focal and ipsilateral off-target fluorescence dimming and bilateral off-target brain activation, can be observed. The dimming partially countered the ipsilateral activation, thereby making it appear weaker than the contralateral side. The US target zone is shown as a black circle. The black polygonal dots represent the boundaries of the TPX windows.

(C) Focal ipsilateral calcium responses returned to the baseline (n=4 animals for each group). No evoked calcium response was observed during the entire 150 s period following the fluorescence dimming after the FUS onset. The interstimulus interval in these experiments was 155 s, consisting of a 5 s baseline recording and a 150 s recording after the FUS onset.

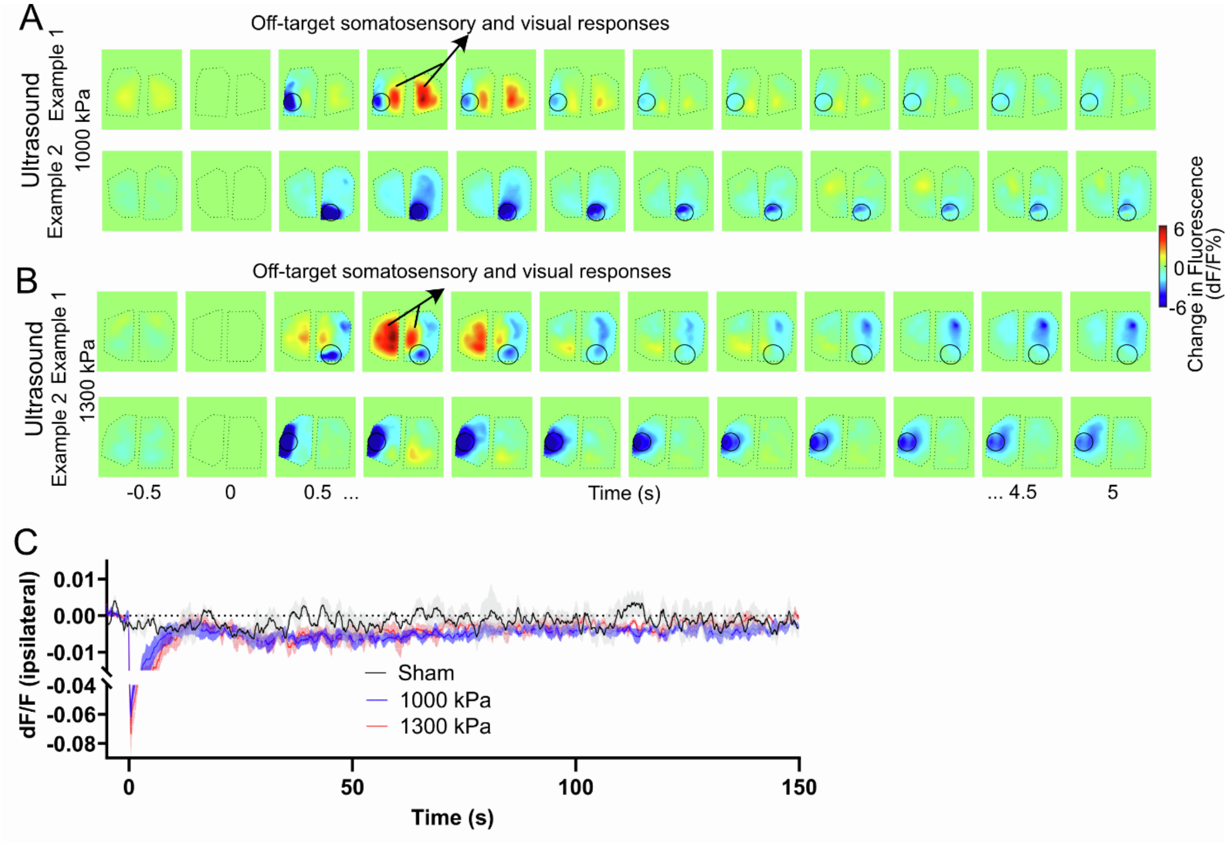

**Figure S4. FUS elicits auditory confounds in normal hearing TPX mice but not deafened TPX mice (Related to Figures 4 and 6, and Table 1).**
(A) A representative cortical map of extensive cortex wide brain activation in the normal hearing mouse through the TPX window in response to continuous ultrasound (916 kHz, 500 ms, pressure at 100, 400, 700, and 1000 kPa). The focus was on the left visual cortex.
(B) A representative cortical map of eliminated off-target brain activation to pulsed ultrasound (270 kHz, Parameter set 1 in Table 1) in the deafened mouse. The focus was on the right visual cortex.

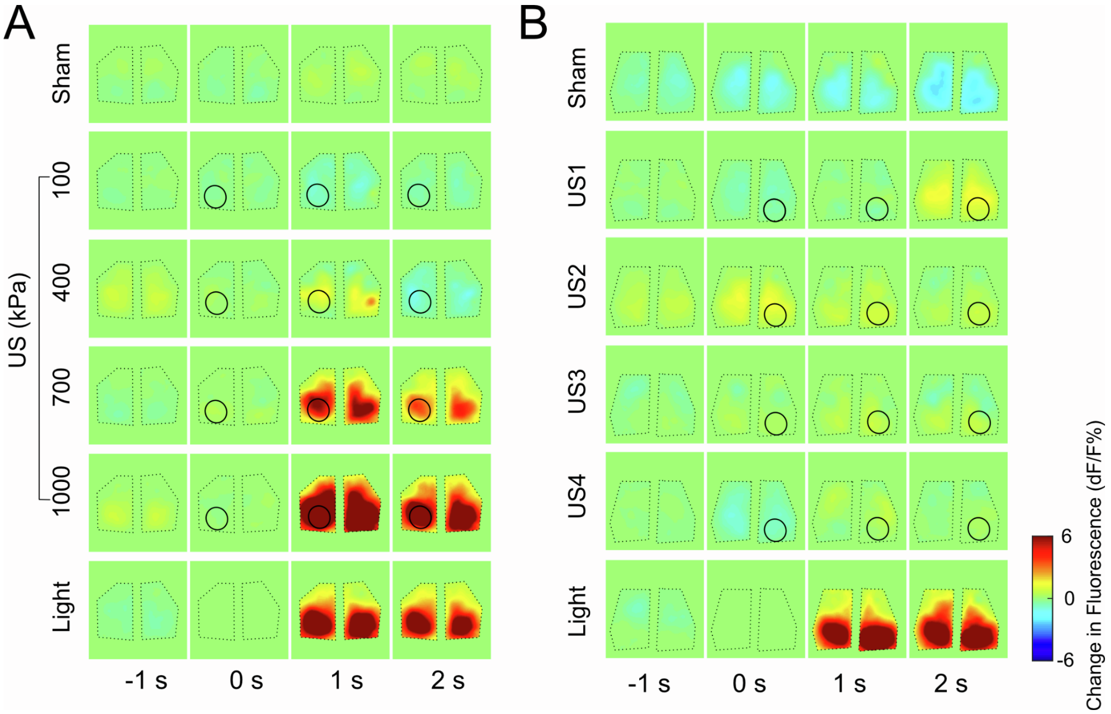


**Figure S5. Temperature increases at the brain surface under the TPX window during UNM (Related to Figure 4).** (A) Peak temperature increases were 0.11 ± 0.01, 0.80 ± 0.04, 2.41 ± 0.14, 5.37 ± 0.55, 10.23 ± 0.54 ℃ (n = 3 animals, 10 trials for each pressure per animal) for 100, 400, 700, 1000, and 1300 kPa, respectively, which were greater than the mean plus three times the standard deviation of the baseline. Bar graph values represent mean ± SEM. (B) The temperature change over a prolonged duration of 150 seconds was plotted for pressures of 1000 kPa and 1300 kPa (n = 3 animals, 5 trials per animal, mean ± SD for better visualization), respectively. The onset of FUS was at 0 s. After 50 seconds, the temperature increase dropped to 0.06 and 0.10 °C for 1000 and 1300 kPa, respectively. The sampling rate of the thermistor was 2 Hz, and each black data point was the average of ten acquisitions at an interval of 10 ms.

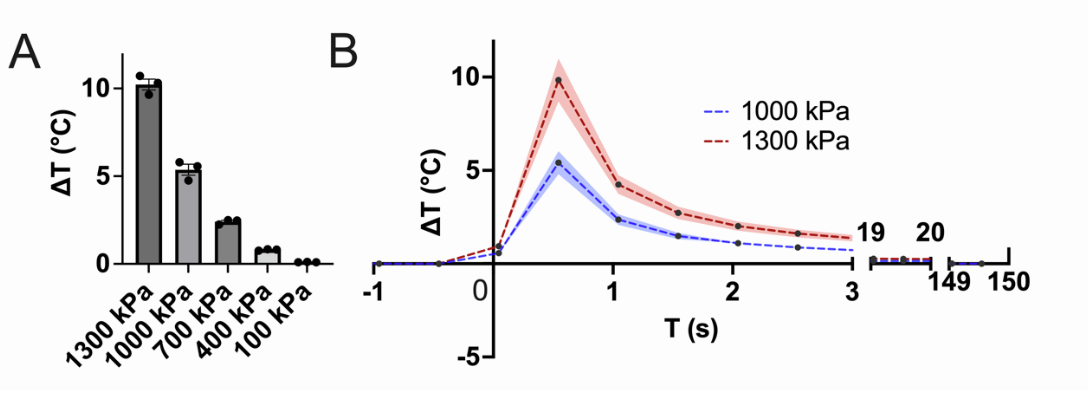

**Figure S6. 916 kHz ultrasound stimulation to the cultured neurons (Related to Figure 4).** (A) Time lapse calcium images of GCaMP6f neurons before and after stimulation. (B) Calcium responses (mean trace is solid, and SEM is shaded.) and (C) quantification of the response as function of ultrasound intensity (mean ± SEM, n = 3 (0 MPa), 5 (0.4-1.6 MPa) independent experiments each, Tukey's multiple comparison test after one-way ANOVA, $p < 0.0001$).

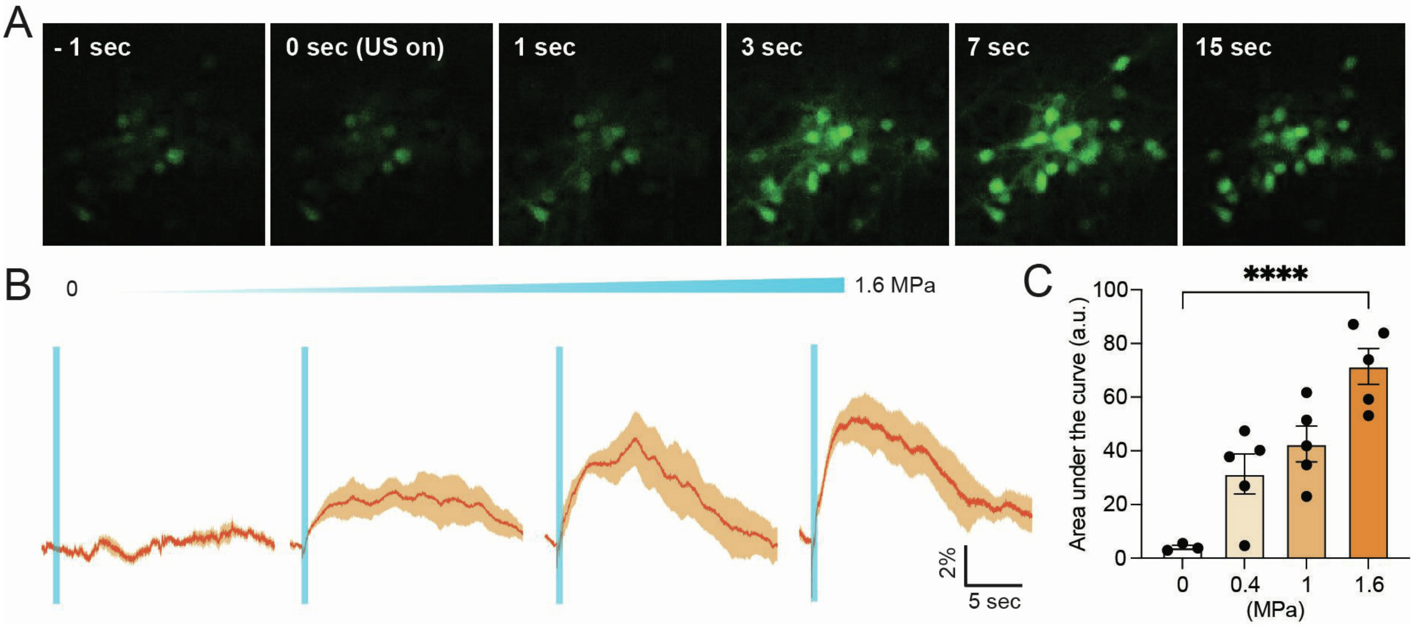

Supplement: Document S1. Figures S1–S6 [file mmc1.pdf]
